# Large-scale monitoring of effects of clothianidin-dressed oilseed rape seeds on pollinating insects in Northern Germany: effects on red mason bees (*Osmia bicornis*)

**DOI:** 10.1007/s10646-016-1729-4

**Published:** 2016-10-05

**Authors:** Britta Peters, Zhenglei Gao, Ulrich Zumkier

**Affiliations:** tier3 solutions GmbH, Leverkusen, Germany

**Keywords:** Solitary bees, *Osmia*, Seed treatment, Plant protection products, Neonicotinoids

## Abstract

The aim of this study was to investigate the effects of Elado® (10 g clothianidin & 2 g beta-cyfluthrin/kg seed)-dressed oilseed rape on the development and reproduction of mason bees (*Osmia bicornis*) as part of a large-scale monitoring field study in Northern Germany, where oilseed rape is usually cultivated at 25–33 % of the arable land. Both reference and test sites comprised 65 km^2^ in which no other crops attractive to pollinating insects were present. Six study locations were selected per site and three nesting shelters were placed at each location. Of these locations, three locations were directly adjacent to oilseed rape fields, while the other three locations were situated 100 m distant from the nearest oilseed rape field. At each location, 1500 cocoons of *O. bicornis* were placed into the central nesting shelter. During the exposure phase, nest building activities and foraging behaviour were assessed repeatedly. Cocoons were harvested in autumn to assess parasitization and reproduction including larval development. The following spring, the emergence of the next generation of adults from cocoons was monitored. High reproductive output and low parasitization rates indicated that Elado^®^-dressed oilseed rape did not cause any detrimental effects on the development or reproduction of mason bees.

## Introduction

Pollination is one of the most essential ecosystem services provided by nature not only to wild plant species, but also for a number of arable crops (Klein et al. 2007). While domesticated honey bees (*Apis mellifera*) are traditionally thought of as the economically most important pollinator in crop monocultures, recent research indicates that wild bee pollinator species can be equally important (Garibaldi et al. 2013). Solitary bees are able to provide pollination services in certain crops with similar or sometimes superior level of efficiency (Bosch et al. 2006). In this regard, it has been shown that they can replace honey bees (Winfree et al. 2007) or act synergistically with them (Garibaldi et al. 2013; Brittain et al. 2013). However, several studies imply that populations of pollinating insects decline (Kearns et al. 1998; Biesmeijer et al. 2006; Potts et al. 2010). As one of the factors responsible for the observed decline the intensification of agriculture was identified, which might also endanger pollination services (Goulson et al. 2015).

In Central Europe, a crop of high economic value is oilseed rape (OSR). Mass-flowering OSR fields were found to have a strong positive influence on the abundance of generalist solitary bees (Holzschuh et al. 2013) and bees of the genus *Osmia* have been shown to be efficient pollinators of OSR (Jauker et al. 2012a). Furthermore, several studies indicated that high amounts of OSR at the landscape scale have positive effects on solitary bees nesting in semi-natural habitats (Jauker et al. 2012b; Diekötter et al. 2014). However, foraging in agriculturally managed fields also enhances the risk of pollinating insects to be exposed to PPPs, which might potentially harmful. Unmanaged (wild) pollinators are considered to be more vulnerable than honey bees due to different foraging behaviour and the fact that nesting sites cannot be moved or covered during PPP applications (Scott-Dupree et al. 2009), which is mostly relevant for spray applications.

One class of PPPs commonly used in OSR are neonicotinoids. Formulations containing neonicotinoids are often used as seed treatment; their active substances are systemically taken up by the plants and distributed to all tissues (Elbert et al. 2008). The use as a seed dressing reduces the risk for non-target organisms, because fewer applications and lower rates are used than in foliar spray applications. However, concerns have been raised about the exposure of flower visiting insects due to the potential presence of the substances in nectar and pollen (Blacquière et al. 2012). Due to these concerns the use of the three neonicotinoids imidacloprid, clothianidin and thiamethoxam was temporarily banned in the European Union in crops attractive to bees (European Commission 2013) to allow for more studies on potential side effects on pollinators.

Despite their ecological value and their potential as pollinators in certain crops, solitary bees were not part of the testing regime routinely used for the registration of PPPs. It should be noted that the toxicity of products may vary between *Osmia* and *A. mellifera* (Ladurner et al. 2005; Biddinger et al. 2013). Also, differences in life history strategies and foraging behaviour may alter the risk under field conditions. Only recently have solitary bees as test organisms, with an associated risk assessment, been included in a new regulatory Guidance Document (EFSA 2013) which is not yet implemented. One reason for the delayed implementation is the lack of robust and reproducible test methods, which account for the biology of solitary bees (synchronisation of emergence, feeding behaviour and regime etc.).

Lower tier studies with solitary bees often represent an unrealistic worst case scenario where individual bees are fed with high dose rates of neonicotinoid contaminated food in the laboratory. For instance, a recent laboratory study (Jin et al. 2015) reports detrimental effects on the ability of *Osmia cornuta* to navigate. However, it has to be considered carefully whether sub-lethal effects found in laboratory studies are reproducible under field conditions. Furthermore, there is a debate as to whether rates used in these experiments really represent the exposure in the field (Carreck and Ratnieks 2014). Thus, a key question is how neonicotinoids influence bees in real world agricultural landscapes (Schmuck and Lewis, 2016, this issue). Monitoring studies at the landscape level, however, have only rarely been conducted.

To our knowledge there is only one study that assesses the effects of a neonicotinoid seed treatment on solitary bees in the field. Rundlöf et al. (2015) conducted a field study with spring OSR grown from Elado^®^-treated seeds in Sweden. Their study concludes negative effects for *Osmia* species. However, there are a number of limitations with regards to the methods used and their ability to realistically assess the effect of the pesticide exposure (low number of cocoons, different crop (spring OSR) with considerably different amount of residues).

Here, we tested whether flowering winter OSR grown from seeds treated with the active substance clothianidin has any potential effects on the nest building activity, development and reproduction of mason bees on the landscape level.

## Materials and methods

### Test species

*Osmia bicornis* (red mason bee) (Megachilidae) is the most abundant solitary bee species in Central Europe (Westrich 1989). This species is univoltine and polylectic. Its lifecycle begins in spring when adult males emerge from their cocoons. Males stay close to the nest to wait for females which emerge few days later. After mating, females begin to build and provision their nests. Nests are built in a wide variety of cavities, which are either naturally occurring or artificially provided. Females collect pollen and small amounts of nectar to provision the separated brood chambers. Each of the up to 30 brood chambers per nest contains one egg (Westrich 1989). Fertilized eggs develop into females and are found in the back end of the linear nest, while unfertilized eggs are laid into the front brood chambers and develop into males. Completely filled nests are sealed with mud.

The foraging range of *O. bicornis* is typically less than 600 m (Gathmann and Tscharntke 2002, Zurbuchen et al. 2010). The active period of foraging and nest building occurs between April and June, and lasts from 6 to 8 weeks. Larvae hatch approximately 3 days after egg laying and feed on the stored pollen and nectar. After the metamorphosis of the larvae to the adult, the bees remain in their cocoons during winter and emerge in the following spring.

For this study, 18 000 cocoons were provided by bienenhotel.de (Rostock, Germany).

### Study location and design

**Fig. 1:**
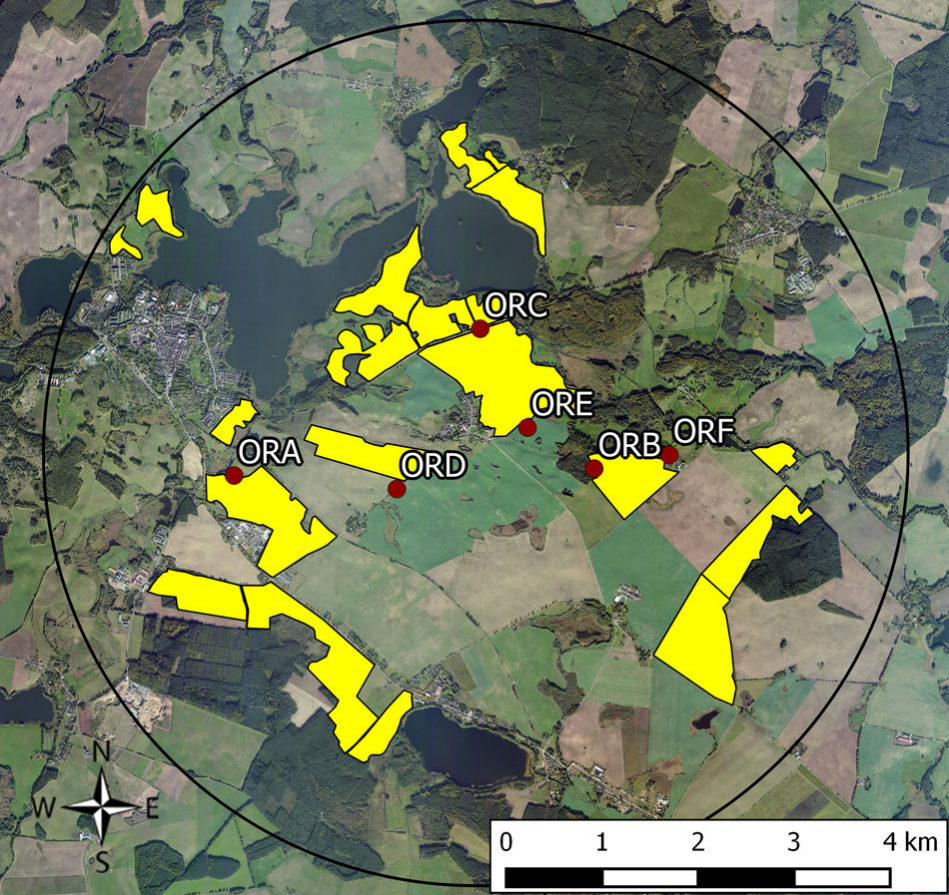
Reference site (fields with untreated seeds) used for the monitoring of effects of flowering OSR grown from clothianidin-dressed seeds on *Osmia bicornis*. Study locations are marked in *blue*; *yellow polygons* indicate the study fields

**Fig. 2:**
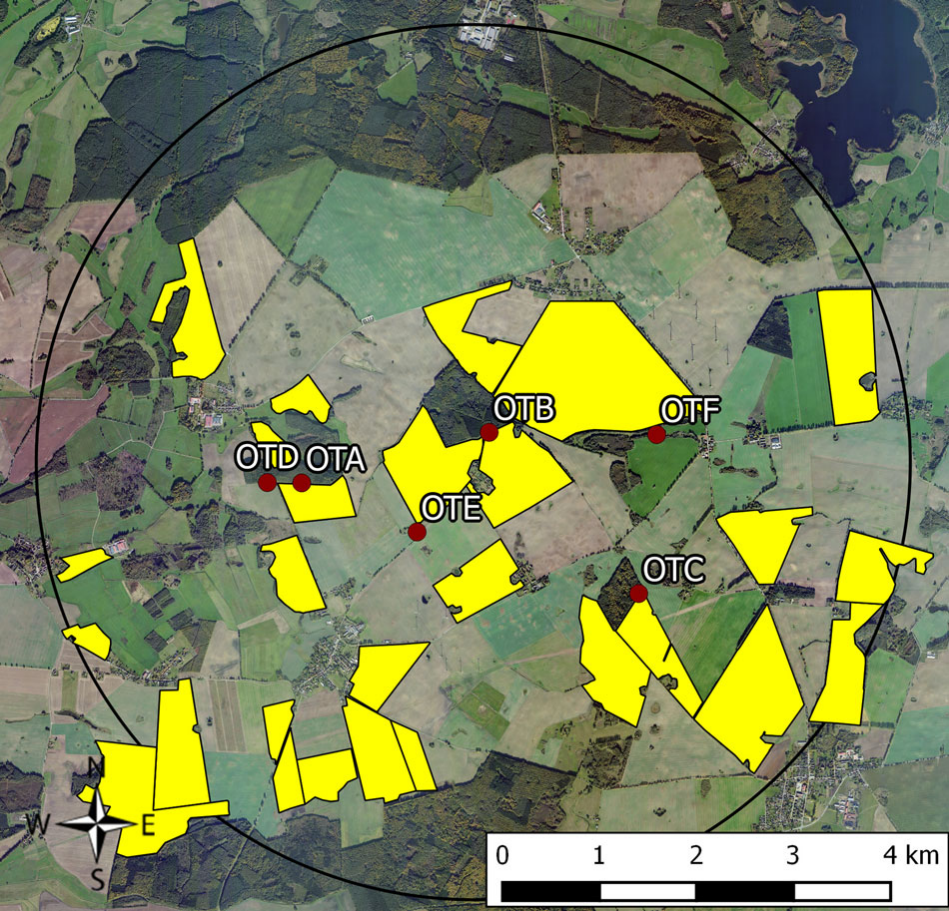
Test site (fields with treated seeds) used for the monitoring of effects of flowering OSR grown from clothianidin-dressed seeds on *Osmia bicornis*. Study locations are marked in *blue*, *yellow polygons* indicate the study fields

The exposure phase of this study was conducted at two neighbouring study sites in the vicinity of Sternberg, Mecklenburg-Vorpommern in Northern Germany. Each study site covered an area of approximately 65 km^2^ with a diameter of 9 km. In autumn 2013 (Fig. 1), Elado^®^-dressed OSR seeds were drilled at all study fields at the test site (Fig. 2), whereas Elado^®^-free OSR seeds were drilled at the reference site (Fig. 1). For a detailed description of seed treatment, OSR fields and planting, see Heimbach et al. (2016, this issue).

**Fig. 3:**
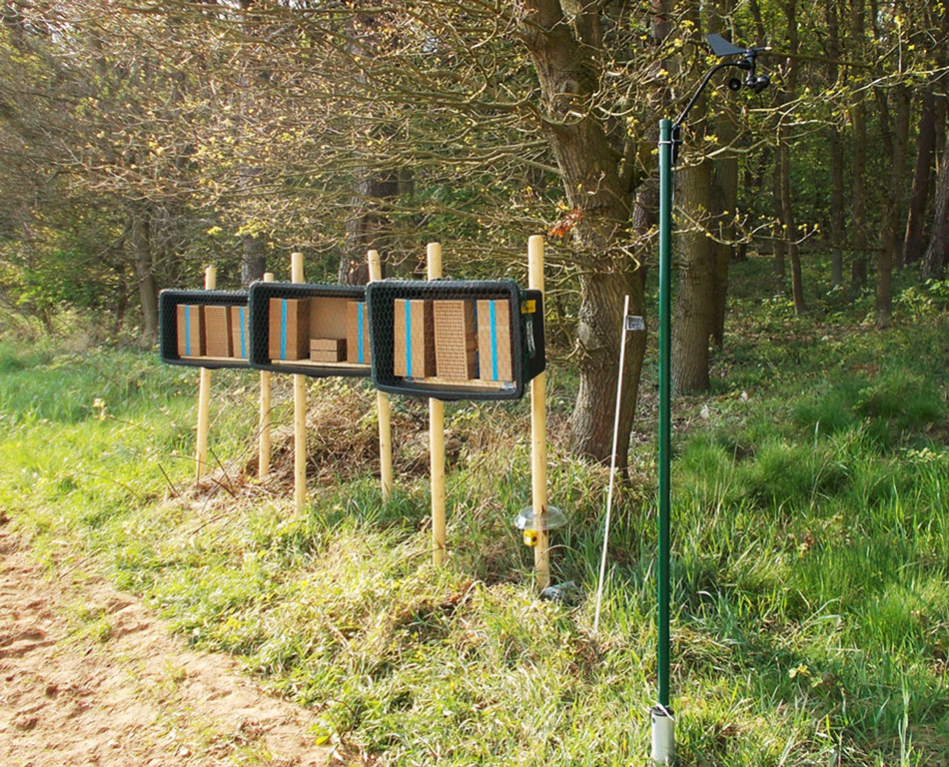
Example of the setup of nesting blocks at the study locations used for the monitoring of effects of flowering OSR grown from clothianidin-dressed seeds on *Osmia bicornis*. The nesting shelter in the middle contains the cardboard boxes with cocoons

Six study locations were established at the inner cores of the test site and reference sites to prevent mason bees to forage outside the study sites. Three out of the six study locations were established at the edge of OSR fields, while the other three were situated 100 m distant from the nearest OSR field. At each study location, three nesting shelters (containing up to three nesting blocks) were established in front of a hedge or forest south-east-facing to be protected against wind and rain and exposed to direct sunlight. Each nesting shelter consisted of a plastic container fixed to two wooden stakes (see Fig. 3). The opening of the shelter was covered with chicken wire to prevent predation by birds. Nesting blocks were obtained from bienenhotel.de (Rostock, Germany). Their special design allowed a non-destructive sampling of pollen as well as the later harvest of cocoons: Nesting blocks consisted of a stack of several fibre boards each drilled with semi-circular nesting tunnels, which were closed on top by the overlying board. Each board contained 10 rows of nesting tunnels arranged in parallel (8 mm in diameter). Boards were lashed together with a strap so that they could be removed and access to the brood chambers was possible (see Fig. S1 Supplementary material).

Two perforated cardboard boxes containing 1500 cocoons (sex ratio male:female 6:5) and in total eight nesting blocks were placed within the three shelters at each location, providing a total of 1600 nesting holes per study location. In each nesting shelter, one nesting block was placed facing south-east, while the two other blocks were placed in a north-east direction. This setup was chosen because of the behaviour of female mason bees when searching for nesting sites: they would be attracted by the south-east-facing blocks as these are clearly visible and then explore the more sheltered north-east-facing blocks which usually were preferred for nesting.

The exposure phase started at the beginning of OSR full flowering (63–65 on the extended BBCH-scale) on 21 April 2014 with the placement of the cocoons (Day After Placement 0). One day after the last assessment of nest occupation at the end of OSR flowering (DAP 32, 23 May 2014, BBCH 74–79), the opening of the nesting shelters was covered with gauze to avoid further nest building activities. Subsequently, on DAP 35, nesting blocks were removed from the study locations and transferred to a sheltered place (an agricultural warehouse) to avoid predation or parasitism.

### Assessments during the exposure phase

Emergence success from the initially provided cocoons was assessed by counting all empty cocoons in the cardboard boxes. This was done twice during the study in order to determine the potential nest building activities.

Nest building activity was observed after sunset, when female mason bees had returned to their nesting holes for the night. Each nesting hole was illuminated with an electric torch and the presence of bees was recorded. In order to determine the start of nest building activities, the first assessment took place on DAP 3. Since there were no females present in 50 % of all study locations (reference and test site), the procedure was repeated the following days until females were found at 75 % of all study locations. After this date (DAP 5) assessments were continued on a weekly basis. In addition to the observation whether females are present, the number of nesting holes sealed with mud was also counted per nesting block.

In order to assess foraging behaviour in nesting cavities, pollen was collected from brood cells. Pollen for the composition analysis was sampled twice at every study location during OSR flowering (DAP 12–13 and 19–20). Nesting blocks were opened for the pollen sampling. 10 subsamples were taken from each nesting block from different nesting holes, these subsamples were pooled. Pollen was only sampled from yet incomplete cells, which were unsealed and did not contain any egg. This procedure was chosen to ensure that all sampled pollen were from roughly the same time and in order not to destroy any finished brood cells, which would have had a negative influence on the outcome of reproduction. Samples were taken from all nesting blocks except for two nesting blocks at study location ORB that did not yield sufficient amounts of pollen during the first sampling event. A total of 190 samples were collected (96 samples per sampling event, 8 nesting blocks per location, and 6 locations at 2 sites, minus two samples as mentioned). Pollen samples were stored deep frozen (−18 °C) until required for microscopical evaluation.

Pollen for residue analysis was sampled likewise once per study location during OSR blossom. The pollen samples for residue analysis were shipped deep frozen on dry ice to Eurofins Agroscience Services Chem GmbH, Hamburg, Germany. Analyses of pollen samples were based on the multi-residue sample preparation technique QuEChERS. The clothianidin residue content was analysed by LC-MS/MS. The Limit of Quantification (LOQ) was 1.0 µg/kg and the Limit of Detection (LOD) 0.3 µg/kg. For further details see Rolke et al. (2016, this issue).

Temperature, humidity, rain fall and wind conditions were recorded at each location. Additionally, the relative duration of sunshine exposure was recorded. A detailed methodology for weather recordings as well as results are given in Heimbach et al. (2016, this issue).

### Assessments after the exposure phase

**Fig. 4:**
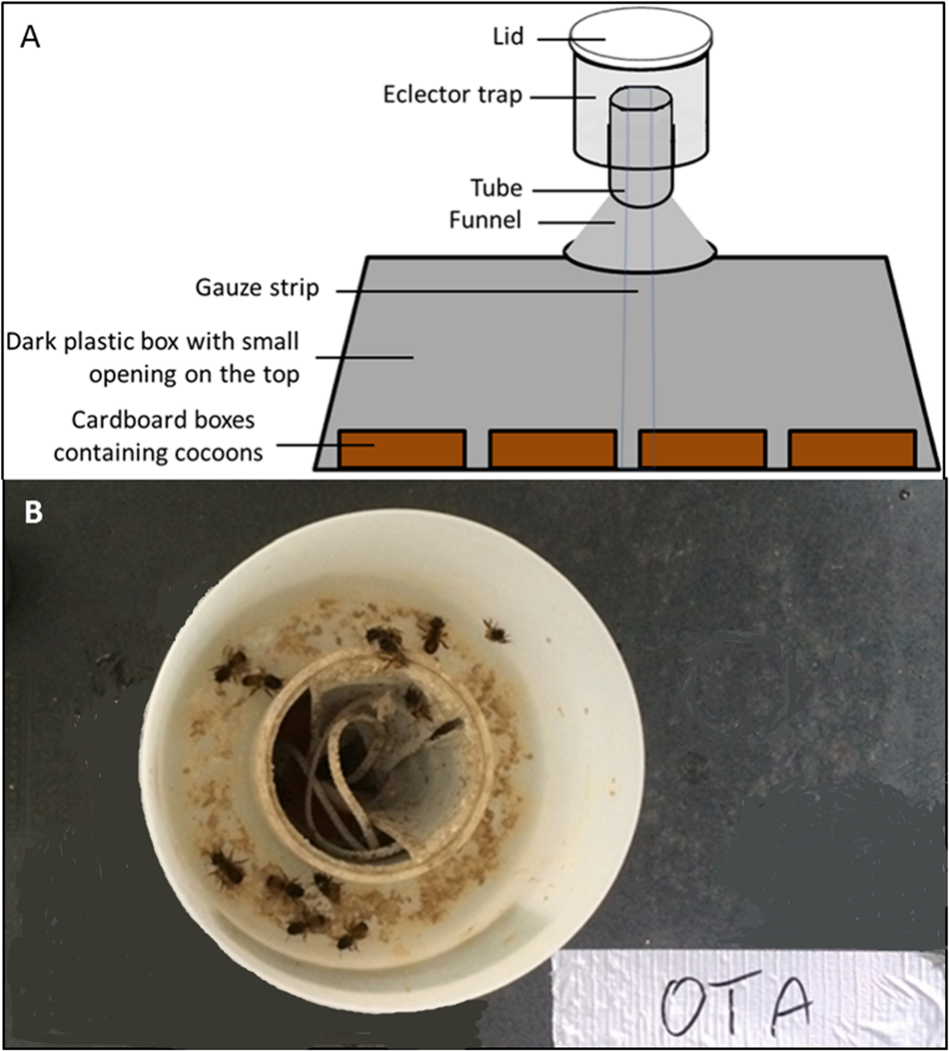
**a** Schematic overview of the contraption used to catch emerging mason bees, **b** photograph from above

In autumn 2014, cocoons of offspring were harvested by dismantling the nesting blocks to evaluate the reproductive output, including an assessment of undeveloped larvae, eggs and parasitism. Subsequently, cocoons were stored in a refrigerator at about 2–4 °C for a duration of approximately 5 months. In spring 2015 (3 March 2015), all cocoons were set up for emergence over a period of approximately 4 weeks. 10 days before the cocoons were removed from the refrigerator, the incubating temperature was gradually raised to 12.5 °C. Cocoons were then placed in a large, black plastic box that was fitted with an “emergence trap” (eclector) (see Fig. 4 for details). Temperature during emergence was about 20 °C.

Emerged male and female bees were counted almost every day until 30 March 2015. Prior to counting, bees were anaesthetised with ether and subsequently released. The number of cocoons that remained closed after 4 weeks were counted and sliced with razor blades to check for undeveloped bees or parasites. During this assessment several bees were found to be alive in the cocoons, but these individuals were not counted as having successfully emerged because it was not clear whether these bees would have managed to emerge from their cocoons.

### Statistical evaluation

Generalized linear mixed models (GLMMs) and Generalized additive mixed models (GAMMs) provide a flexible tool to analyse non-normal and normal data when measurements are not independent due to spatial or temporal grouping. Hence, GLMMs and GAMMS were applied to study the fixed effects of treatment and environmental conditions, while the study location and the individual nesting block were included as random effects. In addition Beta Regression Models were used.

Poisson lognormal GAMMs were used to model the relationship between the number of nesting females and other covariables including the DAP, distance to OSR fields, rotation of nesting block and weather covariables. Study location C at the reference site was identified to differ in the development of nesting females from other locations, because differences in the weather and the combination of a high density and great height of OSR plants at a very short distance from the nesting shelters obstructed access for the mason bees. For the pollen composition data, a beta regression model was fitted to the relative amount of OSR. Poisson GLMMs with observational level random effects and zero-inflated negative binomial GLMMs were fitted to the count data of reproduction endpoints.

Statistical evaluation was conducted with the statistical software package “R” (version 3.0.1, R Development Core Team, Vienna, Austria, 2013). GLMMs were fitted to the data by using the packages “lme4” (Bates et al. 2015) and “nlme” (Pinheiro and Bates 2015). For multiple comparisons of parameters the package “multcomp” (Hothorn et al. 2008) was applied.

The minimum detectable difference (MDD) concept has been developed as an indicator of the power of a test *a posteriori* for aquatic mesocosm/microcosm studies (Brock et al. 2015). However, its calculation depends on the statistical analyses (or tests) applied to analyse the data. The calculation of the MDD for the monitoring study extended the MDD concept to suite the mixed model approach. Augmented predicted confidence intervals were used as the basis for the derivation of the MDD and MDD%.

## Results

### Emergence from cocoons released at the field sites

Emergence of *O. bicornis* started soon after the cocoons were placed in the field: The first males emerged from the cocoons as early as DAP 0 at all study locations, followed by the first females on DAP 3. Nest building started shortly afterwards so that the regular assessments were initiated. On DAP 28, on average 91 % of the released bees had emerged at all study locations (range 87–94 %). The emergence rate did not differ between the reference (91.6 ± 3.2 %, mean ± SD) and the test site (90.0 ± 2.7 %).

### Nest building activities

**Fig. 5:**
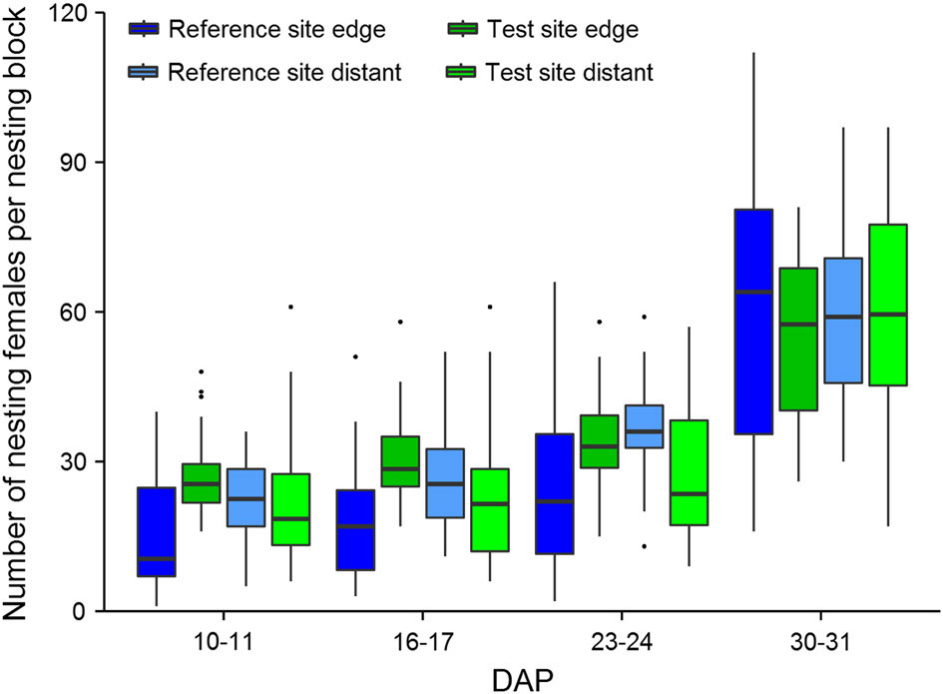
Number of nesting female *Osmia bicornis* per nesting block. Nesting blocks were either situated in a landscape with OSR fields treated with clothianidin seed dressing (test site) or untreated fields (reference site) and placed at the edge of the fields (edge) or ca. 100 m distant from the fields (distant). DAP: Days after Placement. Assessments were conducted over 2 days. Hardly any nesting activities were observed before DAP 10–11. Study location ORC from the reference site was excluded as an outlier (see text for details). The upper and lower hinges of the boxplot correspond to the first and third quartiles (the 25th and 75th percentiles). Whiskers extend from the highest value to the lowest value within 1.5 times the interquartile range. Data beyond the end of the whiskers are plotted as points

Female mason bees accepted the nesting blocks at all study locations. The number of nest building females increased consistently over the course of the study period (despite considerable variability caused by natural fluctuations) (Fig. 5). Except at study location C of the reference site the number of nesting females deviated from the pattern seen at the other study locations from DAP 16 onwards. During the course of the study the OSR at this location grew to exceptional heights (up to 1.9 m) and also very close to the shelters obstructing the access for female mason bees to the nesting shelters. Since the final number of nesting females at this study location was more than twice the standard deviation below the mean it was regarded as an outlier and excluded from further analysis (Table S1, Supplementary material).

Statistical analysis revealed no significant influence of either the treatment or the distance to the OSR fields on the number of nesting mason bees (Tables 1–5). However, the mean sunshine duration was shown to have a positive influence. This influence is underlined by the fact that the increase in the number of nesting females was rather slow at the beginning of the study when the weather in the study area was cold and rainy.

**Table 1:** Statistical significances of the influence of different factors on nesting females and sealed nesting holes

	Nesting females	Completed nesting holes
Intercept	0.83 ± 0.18***	−7.73 ± 0.57***
Treatment	0.18 ± 0.09	1.34 ± 0.31***
Distant to OSR (100 m)	−0.10 ± 0.10	0.88 ± 0.33**
Temperature sum		0.04 ± 0.01***
Wind speed sum	−0.01 ± 0.01	−0.07 ± 0.02***
Mean sunshine duration	1.14 ± 0.22***	0.71 ± 0.73

Nesting blocks were either situated in a landscape with OSR fields treated with clothianidin seed dressing (test site) or untreated fields (reference site) and placed at the edge of the fields (edge) or ca. 100 m distant from the fields (distant). Summary of Poisson lognormal GAMM/Poisson GLMM results. Study location ORC (reference site) was excluded from the evaluation as an outlier (for details, see text). The intercept is the estimated mean value of the dependent variable, when all continuous variables are held at 0 and all categorical variables are held at their baseline levels. Positive values indicate positive interaction, negative values indicate negative interaction. Given are parameter estimates ± standard deviation

***p < 0.001; **p < 0.01; *p < 0.05

**Table 2:** Statistical significances of the influence of the treatment for the relative amount of *Brassica napus* (OSR) pollen in brood cells (beta regression model) of *Osmia bicornis*

	Amount of OSR pollen
Intercept	−1.69 ± 0.09***
Treatment	−0.50 ± 0.10***

Nesting blocks from which pollen was collected were either situated in a landscape with OSR fields treated with clothianidin seed dressing (test site) or untreated fields (reference site) and placed at the edge of the fields (edge) or ca. 100 m distant from the fields (distant). The intercept is the estimated mean value of the dependent variable, when all continuous variables are held at 0 and all categorical variables are held at their baseline levels. Positive values indicate positive interaction, negative values indicate negative interaction. Given are parameter estimates ± Standard deviation

***p < 0.001; **p < 0.01; *p < 0.05

**Table 3:** Statistical significances of the influence of different factors on larval development of *Osmia bicornis*

	Development	
	Undeveloped eggs	Undeveloped larvae
Intercept	1.46 ± 0.32***	2.71 ± 0.20***
Treatment	−1.17 ± 0.44**	−0.75 ± 0.26**
Distant to OSR (100 m)	0.38 ± 0.24	0.26 ± 0.15
Temperature sum	−0.78 ± 0.32*	−0.38 ± 0.18*
Humidity sum	−0.35 ± 0.18	−0.39 ± 0.10***
Wind speed sum	−0.26 ± 0.13*	−0.33 ± 0.08***
Mean sunshine duration	0.34 ± 0.18	0.65 ± 0.11***

Nesting blocks were either situated in a landscape with OSR fields treated with clothianidin seed dressing (test site) or untreated fields (reference site) and placed at the edge of the fields (edge) or ca. 100 m distant from the fields (distant). Summary of Poisson lognormal GLMM results. Study location ORC (reference site) was excluded from the evaluation as an outlier (see text for details). The intercept is the estimated mean value of the dependent variable, when all continuous variables are held at 0 and all categorical variables are held at their baseline levels. Positive values indicate positive interaction, negative values indicate negative interaction. Given are parameter estimates ± standard deviation

***p < 0.001; **p < 0.01; *p < 0.05

**Table 4:** Statistical significances of the influence of different factors on emergence of *Osmia bicornis*

	Emerged individuals		Undeveloped individuals		
	Males	Females	Males	Female	Pupae
Intercept	4.14 ± 0.14***	4.47 ± 0.17***	3.69 ± 0.11***	4.54 ± 0.07***	4.28 ± 0.08***
Treatment	0.41 ± 0.18*	0.26 ± 0.21	−0.21 ± 0.15	−0.59 ± 0.11***	−0.33 ± 0.12**
Distance to OSR (100 m)	−0.02 ± 0.18	0.14 ± 0.21	0.25 ± 0.14	−0.70 ± 0.11***	0.27 ± 0.10**

Nesting blocks from which cocoons were harvested were either situated in a landscape with OSR fields treated with clothianidin seed dressing (test site) or untreated fields (reference site) and placed at the edge of the fields (edge) or ca. 100 m distant from the fields (distant). Summary of Poisson GLMM Results. Study location ORC (reference site) was excluded from the evaluation as an outlier. The intercept is the estimated mean value of the dependent variable, when all continuous variables are held at 0 and all categorical variables are held at their baseline levels. Positive values indicate positive interaction, negative values indicate negative interaction. Given are parameter estimates ± standard deviation

***p < 0.001; **p < 0.01; *p < 0.05

**Table 5:** Summary table of minimum detectable differences (MDDs), relative MDDs (MDD%), and relative differences (%) for various measures of mason bee development and reproduction

Assessment	Variables	MDD	MDD%	Difference (%)
Development of mason bees	Nesting females (DAP 10–31)	3.6–29.1	26.6–35.9	+32.6
	Nesting females (DAP 3–31)	0.0–19.2	24.8–71.0	+19.4
	Completed nesting holes	0.0–22.0	53.7–64. 9	+282.0
Larval development	Undeveloped eggs	4.1–6.6	74.1–78.9	−74.8
	Undeveloped larvae	5.4–6.6	34.6–36.7	−52.6
Emergence	Emerged males	0.0–62.6	31.2–39.5	+6.1 ± 30.1
	Emerged females	0.1–22.4	27.5–34.0	+21.0 ± 50.2
	Undeveloped pupae	13.3–14.7	14.0–20.2	−27.8 ± 47.2
	Undeveloped males	10.2–10.3	19.9–25.6	−19.2 ± 3.3
	Undeveloped females	8.5–17.9	18.4–19.2	−44.7 ± 101.5

Nesting blocks were either situated in a landscape with OSR fields treated with clothianidin seed dressing (test site) or untreated fields (reference site) and placed at the edge of the fields (edge) or ca. 100 m distant from the fields (distant). Study location ORC (reference site) was excluded from the evaluation as an outlier

Predicted confidence intervals, MDDs, and MDD%s for the number of nesting females were calculated based on both the GAMM fitted to the data from DAP 3 to DAP 31, and the GLMM fitted to the data from DAP 10 to DAP 31. The predicted confidence intervals for the number of nesting females partly overlapped for all DAPs (where calculated) and increased with the DAP. During the first two assessments, the number of female bees was close to zero with a MDD < 1. However, the MDD% can be relatively large because the calculation is carried out by dividing the MDD by a very small reference site mean number. Therefore, the first two assessments were excluded from the evaluation. The MDD% derived from the fitted GAMM from DAP 3 to 31 ranged from 24.8 % to 71.0 %, while the MDD% derived from the fitted GLMM from DAP 10 to 31 ranged from 26.6 % to 35.9 % (Table 5), indicating that the test system would have been able to identify a relatively small treatment effect, if present.

**Fig. 6:**
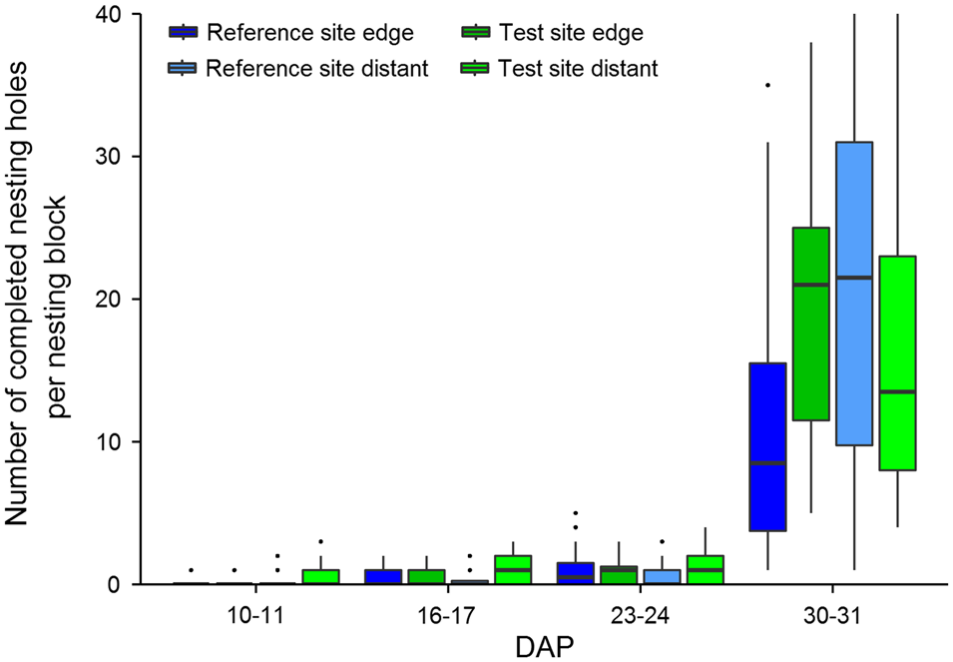
Number of completed nesting holes of *Osmia bicornis* per nesting block. Assessments were conducted over 2 days. Nesting blocks were either situated in a landscape with OSR fields treated with clothianidin seed dressing (test site) or untreated fields (reference site), and placed at the edge of the fields (edge) or ca. 100 m distant from the fields (distant). DAP: Days after Placement. Hardly any nesting activities were observed before DAP 10–11. Study location ORC from the reference site was excluded as an outlier (see text for details). The upper and lower hinges of the boxplot correspond to the first and third quartiles (the 25th and 75th percentiles). Whiskers extend from the highest value to the lowest value within 1.5 times the interquartile range. Data beyond the end of the whiskers are plotted as points

Figure 6 shows the number of nests that were sealed with mud after they were provisioned with pollen and female mason bees had laid an egg inside. Numbers of completed nesting holes were relatively low until the fourth assessment (DAP 23–24), but increased markedly at the end of the study period (on DAP 30/31). At the end of the field phase, numbers of completed nesting holes tended to be lower at the reference site edge locations than at the other study locations. Statistical analysis showed that the number of completed nesting holes were higher at the test site than at the reference site (*p* < 0.001, Table 1). Furthermore, numbers of completed nesting holes were statistically significantly higher at the locations 100 m distant from OSR fields. Weather conditions also significantly influenced the number of completed nesting holes (temperature, humidity, wind speed, Table 1).

Predicted confidence intervals (CI), MDDs, and MDD%s for the number of completed nesting holes were calculated based on the GLMM fitted to the data from DAP 3 to DAP 31. The predicted CIs had almost no overlap and the predicted means in the test site were higher than those in the reference site. The MDD% ranged from 53.7 % to 64.9 % with a general decreasing trend with increasing DAP (Table 5), indicating that the test system was able to identify a moderate treatment effect, if present.

### Pollen composition

**Fig. 7:**
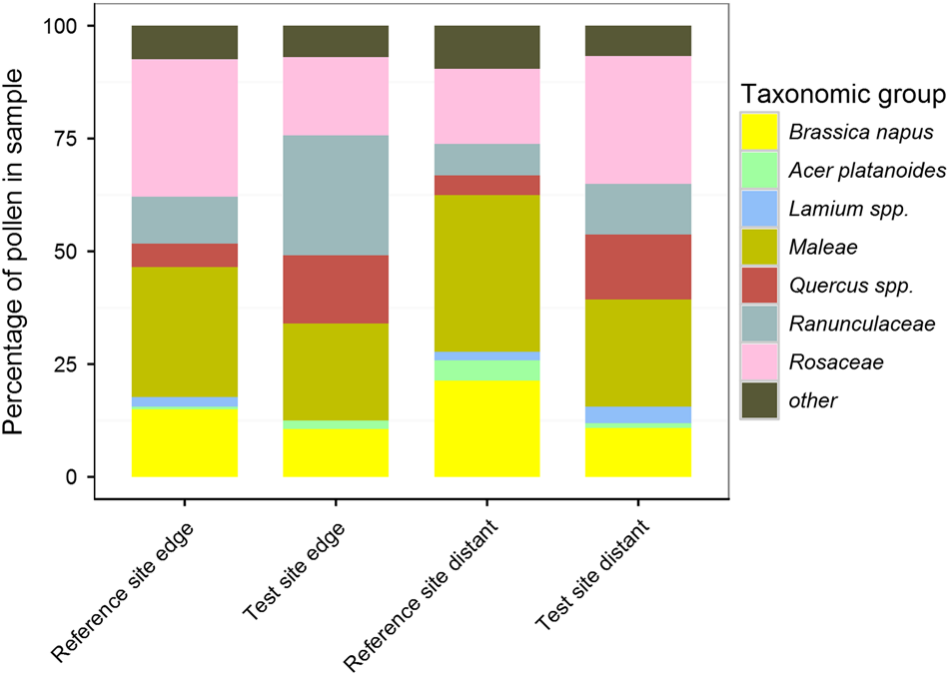
Composition of pollen sampled from nesting blocks occupied by *Osmia bicornis*. Nesting blocks were either situated in a landscape with OSR fields treated with clothianidin seed dressing (test site) or untreated fields (reference site), and placed at the edge of the fields (edge) or ca. 100 m distant from the fields (distant). Mean percentage of various pollen grains in pollen samples from the four experimental groups, reference and test sites. Pollen species representing less than 5 % of the total amount at the reference site were grouped as “other”

The mean (± SD) amount of OSR pollen ranged between 10.6 ± 6.8 % (test site edge) and 21.4 ± 13.2 % (reference site distant) (Fig. 7). Other important pollen sources were Rosaceae (the subfamily Maleae with a maximum amount of 34.7 % and other unspecified Rosaceae with a maximum amount of 30.5 %) and Ranunculaceae (maximum amount of 26.6 %). The proportion of OSR pollen was significantly lower in brood cells at the test site compared to cells at the reference site (*p* < 0.001, Table 2), reflecting the diverse and inhomogeneous landscape (Heimbach et al. 2016, this issue).

### Residues

The clothianidin residues in pollen from nesting cells of mason bees were very low. Only at two study locations at the test site, clothianidin residues were found in quantifiable concentrations (1.1 and 1.7 µg/kg, Rolke et al. 2016, this issue). At the other study locations at the test site the residues were below the LOQ (LOQ = 1.0 µg/kg) and at the reference site below the Limit of Detection (LOD = 0.3 µg/kg). It is not possible to relate the amount of OSR pollen to the residues in the pollen since samples were not collected at the same day. The clothianidin metabolites TZNG and TZMU were not detected in pollen from any study location.

### Reproduction–larval development in autumn

**Fig. 8:**
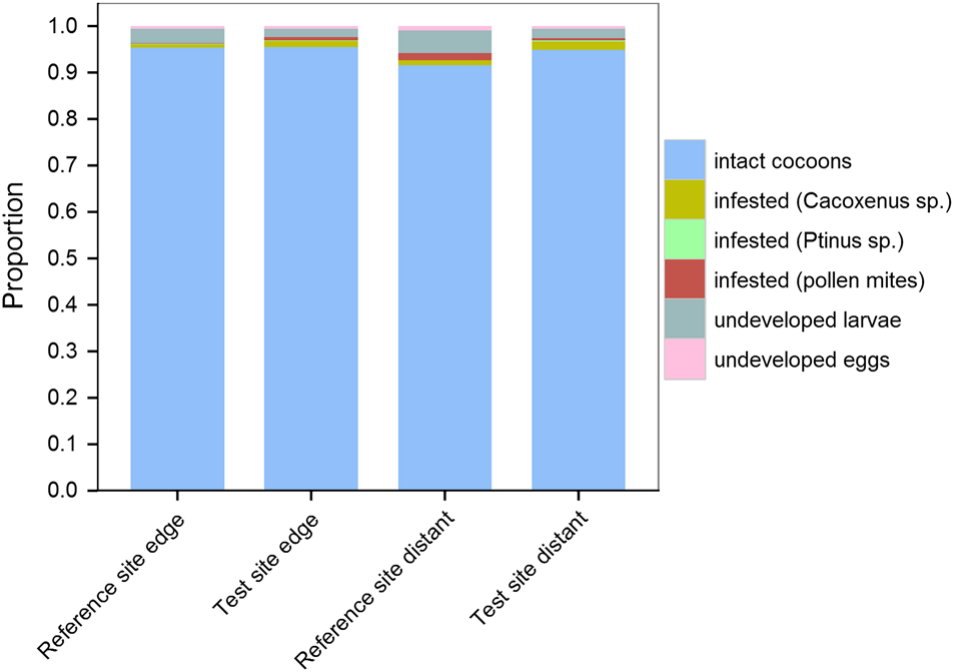
Larval development of *Osmia bicornis*. Nesting blocks were either situated in a landscape with OSR fields treated with clothianidin seed dressing (test site) or untreated fields (reference site) and placed at the edge of the fields (edge) or ca. 100 m distant from the fields (distant). Proportion of intact cocoons in relation to undeveloped larvae/eggs and parasite infested cells as assessed in autumn. Study location ORC (reference site) was excluded as an outlier (see text for details)

Figure 8 shows the proportion of intact cocoons relative to parasitized brood cells and undeveloped larvae/eggs. In total 41 369 intact cocoons were harvested. Regardless of treatment or distance to OSR fields, the proportion of intact cocoons was greater than 0.9. Total mean parasitization rates were 0.97 and 2.62 % for the edge and distant study locations at the reference site, respectively, and 2.12 and 2.47 % for the edge and distant study locations at the test site, respectively. Mean numbers (± SD) are presented in Table S2 in the Supplementary material.

Since the numbers of undeveloped eggs and larvae were significantly lower (*p* < 0.01, Table 3) at the test site, the larval development seemed to be slightly better at the test site.

In general, weather conditions also affected the reproduction endpoints. Temperature and wind speed were negatively correlated with the number of undeveloped eggs and larvae while the relative humidity affected only the number of undeveloped larvae negatively (Table 3). The mean sunshine duration was also an important predictor for the number of undeveloped larvae indicated by a positive relationship with the number of undeveloped larvae. However, since all weather variables were probably correlated these findings have to be treated with caution.

The MDD% for undeveloped individuals in brood cells ranged from 34.6 % to 78.9 % depending on the methodological power of the data generated (Table 5). This indicates that for the reproduction endpoints a small to medium difference could have been determined as statistically significant using the collected dataset, if present.

### Reproduction – emergence after over-wintering

**Fig. 9:**
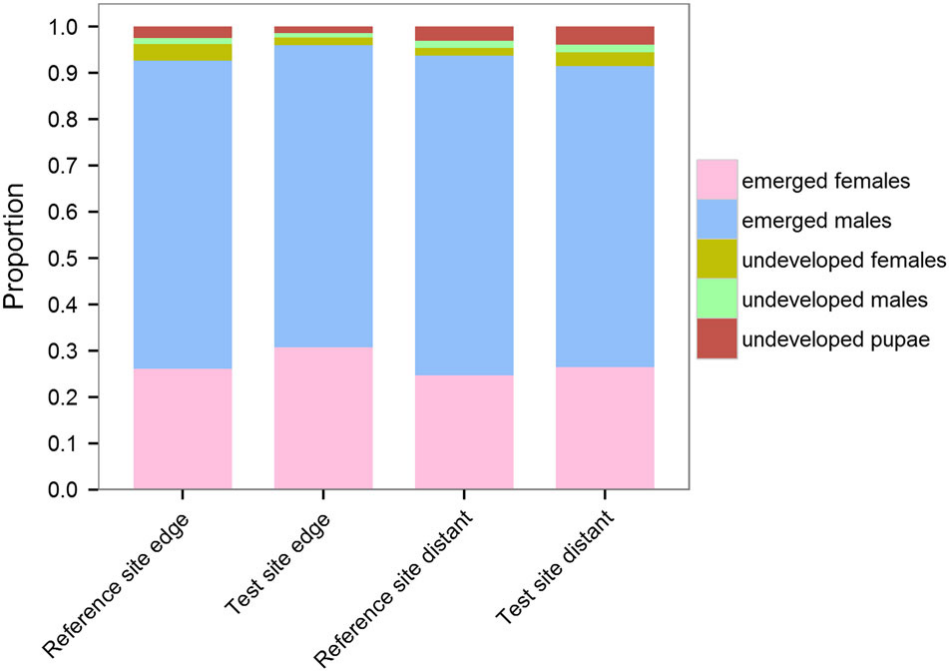
Emergence of *Osmia bicornis*. Nesting blocks were either situated in a landscape with OSR fields treated with clothianidin seed dressing (test site) or untreated fields (reference site) and placed at the edge of the fields (edge) or ca. 100 m distant from the fields (distant). Proportion of females/males successfully emerged in relation to undeveloped individuals as assessed in spring after over-wintering. Study location ORC (reference site) was excluded as an outlier (see text for details)

The evaluation of emergence success after overwintering was based on all intact cocoons harvested in autumn of the previous year which were in total 41 369. From theses cocoons 38 650 individuals (male and female) emerged successfully. The proportion of emerged males and females in relation to undeveloped individuals (male, female, pupae), which remained in the cocoons is presented in Fig. 9. Regardless of treatment or distance to the OSR fields, the proportion of successfully emerged bees was greater than 0.9. Mean numbers (± SD) are presented in Table S3 in the Supplementary material.

Table 4 presents the results of the statistical analysis for the emergence after over-wintering. Significantly more males (*p* < 0.05) emerged from cocoons at the test site compared to the reference site. Significantly higher numbers of undeveloped females (*p* < 0.001) and pupae (*p* < 0.01) were found in the remaining cocoons from the reference site. No parasites were found in remaining cocoons. The distance to OSR fields had no statistically significant influence on the emergence of adult bees. Significantly more undeveloped pupae were recorded for the study locations 100 m distant from OSR fields (*s* < 0.01), whereas more undeveloped females occurred in cocoons from the edge of the OSR study locations (*p* < 0.001).

Predicted confidence intervals, MDDs, and MDD%s for emerged males and females from F1 generation were calculated based on the GLMM fitted to the data from the emergence period. The MDD% for emerged females ranged from 27.5 % to 34.0 %, and for emerged males from 31.2 % to 39.5 % (Table 5). These small MDD% ranges indicate that the experimental design of the study allowed the detection of relatively small treatment effects, if present.

The MDD%s for the different contents of remaining cocoons after emergence of offspring ranged between 14.0 % and 25.6 % for undeveloped individuals (Table 5). This indicates that for the cocoon-related endpoints, a small difference could have been determined as statistically significant using the collected dataset, if present.

## Discussion

The unique reproductive biology and foraging behaviour of *O. bicornis* in the evaluation of potential side effects of clothianidin-treated OSR were the main focus of this study. Mason bees mainly foraged in the surrounding hedges and trees to provision their brood cells, the mean percentage of OSR pollen in the larval food per site was ca. 10–20 %, which is comparable to results found by Holzschuh et al. (2013). As food-generalists (Westrich 1989), the red mason bee forages on a wide variety of plants, but Rosaceae and Ranunculaceae are particularly favoured (Westrich 2014), which was also reflected in our data.

Statistically significant fewer OSR pollen was found in brood cells at the test site. It might be argued that the residues of clothianidin in nectar and pollen had repellent properties, which caused mason bees to reduce their foraging on the treated OSR. However, we did not monitor the foraging activity of *O. bicornis* on OSR fields and the collection of pollen from brood cells is not appropriate to conclude a repellent effect.

There are, however, several circumstances to reject such an effect: The found differences in pollen collected by red mason bees were most likely biased by the comparatively high percentage of OSR pollen found at the distant study locations of the reference site, in combination with a high number of samples that yielded high statistical power to detect even small differences. Furthermore, the variation in the collection of OSR pollen might also be dependent from the availability of alternative foraging resources, which were estimated (Heimbach et al., 2016, this issue) but not controlled for in this study. Therefore, we believe that the statistical significance does not necessarily point to biological relevance.

In conclusion, we could show that *O. bicornis* was exposed to clothianidin via residues found in the pollen. This exposure and therefore the risk is limited due to the foraging behaviour of *O. bicornis*.

For the number of nesting females no factor other than the mean sunshine duration was found to have a significant influence and in particular there was no evidence for any treatment-related effect. However, slightly more completed nesting holes were found at locations 100 m from the OSR fields at both sites. In addition, more completed nesting holes were found at the test site. Nevertheless, this result has to be interpreted with care because of differences in the nest building rates at different study locations. This can originate from mason bees closing their nesting hole after finishing nest building regardless of whether the nesting hole was filled completely with brood cells or not. This means that some mason bees finish their nest building earlier than others. Hence, the number of completed holes is a highly variable factor. As we did not record the number of cocoons per nesting tube, the number of closed nesting holes per se is of limited value to draw any conclusion on treatment-related effects.

The relative duration of sun exposure was the most important weather variable for the development of mason bees, since the number of both nesting females and offspring was significantly positively correlated with the sunshine duration at nesting shelters. The distance of nesting shelters to OSR fields did not have any significant effects on mason bee development. Furthermore, less undeveloped larvae and eggs were found at the test site in comparison to the reference site. This effect is not considered treatment-related, but might be due to variation in the microclimatic conditions between the study locations.

The higher proportion of male to female offspring reflected the reproductive strategy of red mason bees as their mating system (described in detail in Seidelmann, 1999) requires more males than females to be present. Nevertheless, statistical analysis showed that significantly more males were produced at the test site in comparison to the reference site. Populations under stress are known to shift their offspring production to a more male biased ratio (Bosch & Kemp, 2002).This shift can also be a reaction to declining foraging efficiency later in the season or a strategy to minimize the risk of parasitism (which increases with time absent from the nest) (Seidelmann 2006). However, parasitization rates below 3 % are low compared to other investigations on parasitization of mason bees (Seidelmann 1999). Furthermore, our data do not necessarily hint to a shift in the production of offspring. While indeed statistically significant more male offspring were found at the test site, also more females were found at the test site, although this result was not statistically significant.

In order to obtain data of ecological relevance, our focus was not on individual mason bees, but to monitor any potential impact on the populations of *Osmia bicornis*. Our data bear no evidence for an adverse effect on the overall fitness of populations of red mason bees. Our results clearly indicate that no adverse effect on nesting activity was caused by the treatment. This is in contrast to a recent study by Rundlöf et al. (2015) who reported a negative impact. While they found that nesting activities of *Osmia bicornis* completely ceased on fields adjacent to spring OSR fields treated with a clothianidin containing formulation, they cannot provide data that connect the observed effect with the exposure to clothianidin. As the mason bees found no nests at all at the treated fields they cannot provide any pollen analysis that could show that mason bees foraged on the treated crop. No assessment of actually nesting females (e.g., counting of females in the tubes in the evening after daily bee flight) of *Osmia* was performed. It would be of interest to know how many of the emerged females started building a nest and to get a feeling if there is an influence of natural occurring populations in the surrounding of the nesting shelters. Problematic is also the low number of mason bees released in the field. Only 27 cocoons (12 female, 15 male) were placed at each field. Since the authors state an average emergence from these cocoons of ca. 0.4 and considering that only females build nests, their data are based on less than five individuals per field. Failure to build nests could also have been caused by failure to mate at such low densities. Furthermore, the study was conducted outside the peak activity of *Osmia bicornis*, therefore, it is possible that it was also outside the flowering period of other floral resources important to mason bees, which also could have influenced nesting success.

Under consideration of all these aspects, we think that the conclusion that their observed effect was treatment-related is a bit biased. Nevertheless, even if the observed effects are real, the amounts of residues found in Rundlöf et al. (2015) are far higher in comparison to ours and other studies, which could provide an alternative explanation for the different outcome. A detailed discussion on this subject can be found in Rolke et al. (2016, this issue).

## Conclusion

As monitoring studies at the landscape level are complex, difficult to conduct and highly resource-intensive they are not regularly performed for the assessment of risk of PPPs to pollinating insect species. However, they provide an assessment that cannot be obtained from lower tier studies, e.g., in relation to realistic exposure levels and the relevance of the measured endpoints.

This is particularly true for mason bees as standardised methods for testing are not available and consequently different methods for the assessment of effects of PPPs may lead to different (even contrasting) results. Furthermore, repeating field and monitoring studies in subsequent years and/or other locations might be desireable in order to address remaining questions. However, a repetition of this monitoring study was not feasible because the logistics and demands of resources of such a big landscape study exceed common practices by far. Alternatively, certain aspects might even be addressed by specially designed lower tier studies.

The results that we obtained by employing appropriate methods under realistic field conditions show no adverse effects on populations of red mason bees (*Osmia bicornis*) in a landscape monitoring study with high proportion of winter OSR fields treated with Elado^®^.

## Electronic supplementary material

Supplementary Information
