# Supplementary material for: Large-scale monitoring of effects of clothianidin-dressed oilseed rape seeds on pollinating insects in Northern Germany: effects on red mason bees (Osmia bicornis)
Source: Ecotoxicology. 2016 Oct 5;25(9):1679–90. doi: 10.1007/s10646-016-1729-4 (PMC5093198; doi:10.1007/s10646-016-1729-4)

# Electronic Supplementary material to

## Large-scale monitoring of effects of clothianidin dressed oilseed rape seeds on pollinating insects in Northern Germany: Effects on red mason bees (*Osmia bicornis*)

Britta Peters[a*], Zhenglei Gao[a], Ulrich Zumkier[a]

[a] tier3 solutions GmbH, Leverkusen, Germany

* Corresponding author

tier3 solutions GmbH, Kolberger Straße 61-63, 51381 Leverkusen, Germany
Email: britta.peters@tier3.de
Tel: +49 2171 55 997 52
Fax: +49 2171 55 997 69

## Supplementary Tables

**Table S1 :** Total numbers of nesting females as recorded on each assessment for each study location. Nesting blocks were either situated in a landscape with OSR fields treated with clothianidin seed dressing (test site) or untreated fields (reference site, locations ORA-ORF) and placed at the edge of the fields (edge, locations OTA-OTF) or ca. 400 m distant from the fields (distant). DAP: Days after Placement

| | Study Location | | | | | | | | | | | |
|---|---|---|---|---|---|---|---|---|---|---|---|---|
| **DAP** | | **Edge** | | | **Distant** | | | **Edge** | | | **Distant** | |
| | **ORA** | **ORB** | **ORC** | **ORD** | **ORE** | **ORF** | **OTA** | **OTB** | **OTC** | **OTD** | **OTE** | **OTF** |
| 3 | 0 | - | 0 | - | 0 | - | 0 | - | - | 0 | 0 | - |
| 4 | 0 | 0 | 0 | 0 | 0 | 4 | 1 | 1 | 0 | 3 | 0 | 1 |
| 5 | 3 | 0 | 8 | 0 | 3 | 4 | 2 | 6 | 0 | 4 | 4 | 3 |
| 10 | - | 51 | 138 | 177 | - | 193 | - | 220 | 194 | - | 255 | 154 |
| 11 | 189 | - | - | - | 181 | - | 238 | - | - | 101 | - | - |
| 16 | - | 79 | 111 | 196 | - | 269 | - | 272 | 229 | - | 281 | 178 |
| 17 | 213 | - | - | - | 175 | - | 230 | - | - | 110 | - | - |
| 23 | - | 86 | 146 | 288 | - | 306 | - | 270 | 254 | - | 325 | 168 |
| 24 | 344 | - | - | - | 277 | - | 287 | - | - | 176 | - | - |
| 30 | - | 517 | 120 | 431 | - | 527 | - | 417 | 425 | - | 522 | 515 |
| 31 | 457 | - | - | - | 453 | - | 484 | - | - | 409 | - | - |

**Table S2:** Larval development of *Osmia bicornis*. Nesting blocks were either situated in a landscape with OSR fields treated with clothianidin seed dressing (test site) or untreated fields (reference site) and placed at the edge of the fields (edge) or ca. 400 m distant from the fields (distant). Mean number (±SD) of intact cocoons, infested cocoons, undeveloped larvae and eggs as recorded in autumn. Study location ORC was excluded as an outlier (see text for details)

| | **Reference edge** | | | **Test site edge** | | | **Reference distant** | | | **Test site distant** | | |
|---|---|---|---|---|---|---|---|---|---|---|---|---|
| | **Mean** | **±** | **SD** | **Mean** | **±** | **SD** | **Mean** | **±** | **SD** | **Mean** | **±** | **SD** |
| intact cocoons | 3189.50 | ± | 836.51 | 4058.33 | ± | 548.54 | 3486.67 | ± | 1415.67 | 3764.67 | ± | 357.64 |
| infested (*Cacoxenus* sp.) | 23.50 | ± | 20.51 | 56.00 | ± | 78.00 | 39.67 | ± | 24.50 | 67.33 | ± | 61.86 |
| infested (*Ptinus* sp.) | 6.50 | ± | 9.19 | 3.67 | ± | 3.51 | 1.67 | ± | 2.89 | 9.00 | ± | 13.89 |
| infested (pollen mites) | 2.50 | ± | 0.71 | 30.67 | ± | 19.63 | 58.33 | ± | 72.75 | 21.67 | ± | 6.66 |
| undeveloped larvae | 105.00 | ± | 86.27 | 75.33 | ± | 31.90 | 183.67 | ± | 75.50 | 79.00 | ± | 18.08 |
| undeveloped eggs | 18.00 | ± | 9.90 | 24.00 | ± | 15.39 | 38.00 | ± | 11.00 | 23.67 | ± | 8.50 |

**Table S3:** Emergence of *Osmia bicornis*. Nesting blocks were either situated in a landscape with OSR fields treated with clothianidin seed dressing (test site) or untreated fields (reference site) and placed at the edge of the fields (edge) or ca. 400 m distant from the fields (distant). Mean number (±SD) of emerged males and females, undeveloped males and females, and undeveloped pupae as assessed in spring after over-wintering. Study location ORC was excluded as an outlier (see text for details)

| | **Reference edge** | | | **Test site edge** | | | **Reference distant** | | | **Test site distant** | | |
|---|---|---|---|---|---|---|---|---|---|---|---|---|
| | **Mean** | **±** | **SD** | **Mean** | **±** | **SD** | **Mean** | **±** | **SD** | **Mean** | **±** | **SD** |
| emerged females | 832.00 | ± | 59.40 | 1247.67 | ± | 17.04 | 860.00 | ± | 346.63 | 997.67 | ± | 186.92 |
| emerged males | 2123.00 | ± | 998.43 | 2646.33 | ± | 573.29 | 2406.67 | ± | 941.11 | 2445.00 | ± | 196.85 |
| undeveloped females | 113.50 | ± | 101.12 | 68.00 | ± | 38.57 | 60.00 | ± | 33.60 | 112.00 | ± | 111.00 |
| undeveloped males | 43.50 | ± | 36.06 | 39.33 | ± | 30.66 | 53.67 | ± | 21.13 | 60.67 | ± | 38.70 |
| undeveloped pupae | 77.50 | ± | 84.15 | 57.00 | ± | 64.86 | 106.33 | ± | 103.37 | 149.33 | ± | 151.13 |

## Calculations of Minimum Detectable Differences (MDDs)

MDDs as proposed by Brock et al. (2014) were developed as a posteriori indicator of a power of a test. However, these calculations depend on the statistical analysis that was used (e.g. two sample t-test). In this study mixed models were used which made an adaption of the formula necessary.

A generalized linear mixed model (Bates et al. 2014) can be expressed by the following equations:

$$Y_i = X_i\beta + Z_i b_i + \epsilon_i$$
$$b_i \sim N(0, D)$$
$$\epsilon_i \sim N(0, \Sigma_i)$$

The implied marginal model is then: $Y_i \sim N(X_i\beta, Z_i D Z_i' + \Sigma_i)$

Assume a general linear hypothesis of the following form is of analysis interest:

$$H_0: \xi = L\beta - \xi_0 = 0 \text{ versus } H_a: \xi \neq 0$$

The test statistic is approximately F-distributed under $H_0$

$$F = \hat{\xi}'(L(\textstyle\sum_i X_i' V_i^{-1} X_i)^{-1} L')\hat{\xi}/(\mathrm{rank}(L))$$

Under the alternative hypothesis $H_a$ the distribution of F can also be approximated by a noncentral F-distribution (Kirk 1995).

Based on the F-distributed test statistic, the confidence interval of ξ can be calculated and the MDD is then half of the confidence interval. However, in many instances such a linear hypothesis cannot easily be compiled because various kinds of tests were applied, including Wald-t and likelihood ratio tests, according to different scenarios. The distributions of the corresponding test statistics are often only known under the null hypothesis. In practice, extensive simulations would be required. Moreover, it is often impossible to transform the test statistic confidence intervals or critical values back to the original scale of the response variable. Hence, the MDD calculated according to the test statistic has no direct biological meaning and conveys no intuitive information on how powerful the conducted tests were.

Furthermore, nonlinear relationships between the response variables and the predictors, interaction of the treatment groups, and the predictors complicate the achievement of an estimate for the treatment effect. In addition, unlike simple multiple comparisons in mesocosm/microcosm studies, multiple covariate variables were involved in our models, like day after placement (DAP), temperature sum, and humidity sum

A practical solution to the above problems is to calculate an MDD analogue based on the prediction confidence intervals (CIs) instead of the test statistic. The aim is to compare the reference group mean $x_0$ with the treatment group mean $x_1$. The mean confidence interval bounds are based on the magnitude of the standard errors $(SE_0,\ SE_1)$ while the calculation for the test statistic for the difference between two means is based on the square root of the sum of squares of the standard errors $\left(\sqrt{SE_0^2 + SE_1^2}\right)$, similar to the two-sample t-test case. Two means are statistically significantly different (with confidence level $\alpha = 0.05$) when $(x_0 - x_1) - 1.96\left(\sqrt{SE_0^2 + SE_1^2}\right) > 0$, that is, when the CI for the difference between the two group means does not contain zero. Two means do not have overlapping confidence intervals if $x_0 - 1.96SE_0 > x_1 + 1.96SE_1$, that is, if the lower bound of the CI for the greater mean (here it is assumed that $x_0$ is greater than $x_1$) is greater than the upper

bound of the CI for the smaller mean (Environment Canada 2005). With some of algebraic manipulation the following can be proved:

The means are significantly different when $(x_0 - x_1) > 1.96\left(\sqrt{SE_0^2 + SE_1^2}\right)$. There is no overlap between CIs when $(x_0 - x_1) > 1.96(SE_0 + SE_1) > 0$. It is always the case that the square root of the sum of squares of two numbers is less than the sum of those numbers, i.e. $\sqrt{SE_0^2 + SE_1^2} < SE_0 + SE_1$. Therefore, as the difference in the means $(x_0 - x_1)$ increases, it becomes significantly different before the two group mean confidence intervals cease to overlap.

The following procedure was conducted to calculate an MDD on the scale of the observed difference in response variables between the reference and the treatment Site.

1. Data augmentation.
   - Use DAP as the primary predictor when it is used as a predictor in the model.
   - For further covariates besides primary predictor used in the prediction model, their average (numeric covariates), most frequent value (categorical covariates), or each category (distance groups, independent sampling events) were used to obtain the predicted values.
2. Calculate estimate for both treatment and reference group at each assessment DAP or all DAPs as well as corresponding standard errors. The method to obtain the prediction confidence intervals and the standard errors are described in various references (Zuur et al 2009, Bolker et al 2009, Buja et al. 2009). The prediction CIs disregard the random effects from study locations because the fixed treatment effect is of main interest. The natural variation in the mason bee development caused by the different locations were captured by the random effect components in the models and were not taken into account in the determination of the treatment related effect.
3. Calculate MDD by $1.96\left(\sqrt{SE_0^2 + SE_1^2}\right)$ at each DAP. It is also convenient to give the MDD as a percentage of the reference means over DAP.
4. A very conservative estimate of the MDD can be obtained by $1.96(SE_0 + SE_1)$.
5. In case of a generalized linear/additive mixed model or a transformation of the outcome variable applied before fitting the model, the MDD is calculated in the response scale by transforming the predicted mean and standard errors using the inverse link function or inverse transformation function.

The above described procedure is not restricted to any particular statistical test and is biologically meaningful. Thus, a consistent method can be used for all conducted analysis on various types of data and the power of the hypothesis testing conducted is intuitively easy to assess.

## Additionally cited references

Bates D, Mächler M, Bolker B, Walker S (2014) Fitting Linear Mixed-Effects Models using lme4. arXiv:1406.5823 [stat.CO]

Bolker BM, Brooks ME, Clark CJ, et al (2009) Generalized linear mixed models: a practical guide for ecology and evolution. Trends in Ecology & Evolution 24:127–135. doi: 10.1016/j.tree.2008.10.008

Brock TCM, Hammers-Wirtz M, Hommen U, et al (2014) The minimum detectable difference (MDD) and the interpretation of treatment-related effects of pesticides in experimental ecosystems. Environ Sci Pollut Res 22:1160–1174. doi: 10.1007/s11356-014-3398-2

Buja A, Cook D, Hofmann H, et al (2009) Statistical inference for exploratory data analysis and model diagnostics. Philosophical Transactions of the Royal Society of London A: Mathematical, Physical and Engineering Sciences 367:4361–4383. doi: 10.1098/rsta.2009.0120

Environment Canada (2007) Guidance document on statistical methods for environmental toxicity tests. Report EPS 1/RM/46. Ottawa

Kirk RE (2012) Experimental Design: Procedures for the Behavioral Sciences, 3rd edition. Pacific Grove, CA: Brooks/Cole Publishing

Zuur A, Ieno EN, Walker N, et al (2009) Mixed Effects Models and Extensions in Ecology with R, 1st edition. Springer, New York

## Supplementary Figure

**Fig. S1:** Nesting blocks used for the experiment monitoring the effects of clothianidin treated OSR on *Osmia bicornis*. **a**: Nesting blocks consisted out of several fibre boards each drilled with a semi-circle so that two boards formed a nesting tunnel. Boards were lashed together with a strap so that they could be removed and access to the brood chambers was possible **b**: Overview of the dimensions of the boards that the nesting blocks consisted of. **c**: Photograph of a board of a dismantled nesting block showing stored pollen and eggs

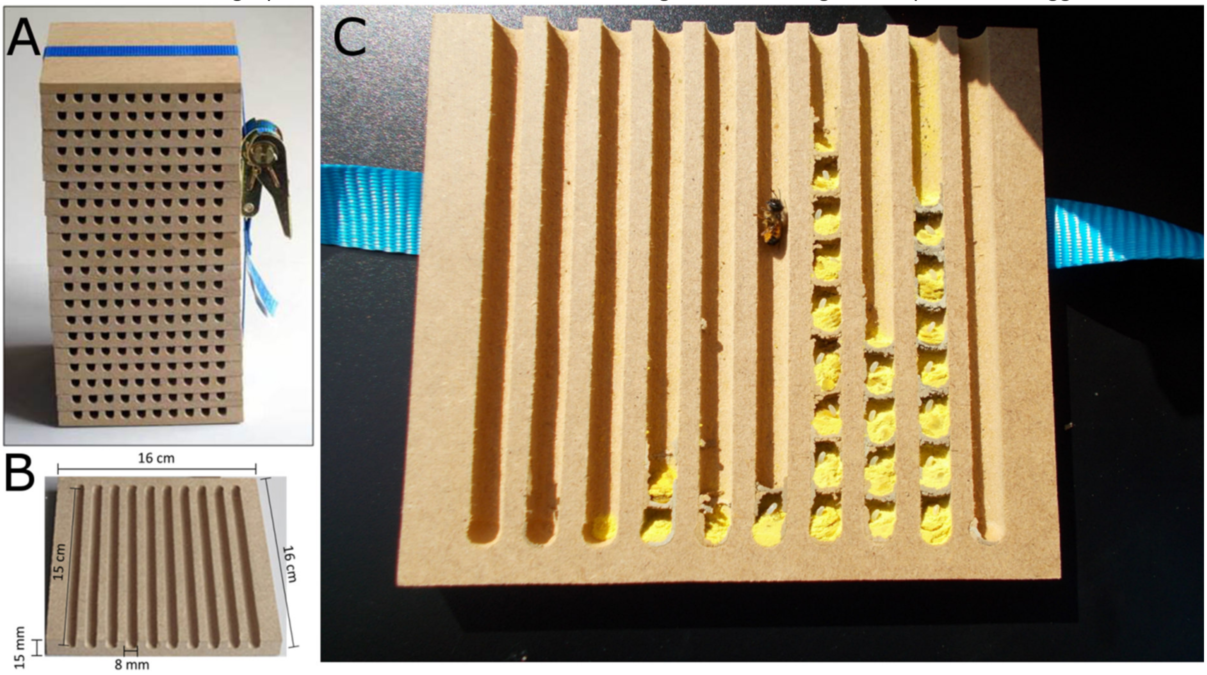

Supplement: Supplementary file 1 — Supplementary Information [file 10646_2016_1729_MOESM1_ESM.pdf]
